# Changes of Cytokines in Children With Tic Disorder

**DOI:** 10.3389/fneur.2021.800189

**Published:** 2022-01-11

**Authors:** Yilin Tao, Peng Xu, Weiyi Zhu, Zhiyue Chen, Xiaohan Tao, Jiajing Liu, Ziru Xue, Tao Zhu, Peifang Jiang

**Affiliations:** ^1^Department of Neurology, Children's Hospital, Zhejiang University School of Medicine, National Clinical Research Center for Child Health, Hangzhou, China; ^2^Department of Pediatrics, Tongxiang First People's Hospital, Tongxiang, China; ^3^School of Mental Health, Wenzhou Medical University, Wenzhou, China; ^4^Department of Pediatrics, Center Hospital of Karamay, Xinjiang, China; ^5^Department of Critical Care Medicine, Sir Run Run Shaw Hospital, School of Medicine, Zhejiang University, Hangzhou, China

**Keywords:** immune, cytokines, tic disorder, Tourette syndrome, neurology

## Abstract

Tic disorder (TD) is a common childhood-onset disease associated with abnormal development of brain networks involved in the motor and sensory processing. The underlying pathophysiological mechanisms in TD are still unclear. An involvement of immune mechanisms in its pathophysiology has been proposed. This study investigates the association between the changes of cytokines and the etiology and development of TD. Different expressions of cytokines in a larger number of samples in our study may provide new insights to the field. The levels of cytokines (IL-2, IL-4, IL-6, IL-10, TNF-α, and IFN-γ) were evaluated in 1,724 patients who were clinically diagnosed with TD from 1 to 17.5 years old and 550 were from 6 months to 14.5 years old in the control group. We assessed the levels of cytokines according to the patient's medication status and the severity of the disease. Of the cytokines we investigated, the serum IL-6 concentration of children with TD was significantly higher than that of the control group, while the levels of other cytokines were lower in TD patients. In the patient group whose YTGSS score ranged from 1 to 9, the IL-4, IL-10, and IFN-γ levels increased in medication group compared to unmedication group. Our data suggested that the cytokines (IL-2, IL-4, IL-6, IL-10, TNF-α, and IFN-γ) may play an important role in the etiology and the severity in TD. Whether drug intervention in the early stage of tic disorder has a better effect on children needs further research.

## Introduction

Tic disorder (TD), is a childhood-onset neuropsychiatric disease, characterized by sudden, rapid, repetitive, unwanted, non-target-oriented non-rhythmic movements or phonic tics. TD includes Tourette syndrome (TS), provisional tic disorder (PTD) and chronic motor or vocal tic disorder (CTD). In TD, 95% of cases occur between the age of 4 and 13 years. The severity of TD may increase over time, peaking around puberty (1). The population prevalence of TD in children was about 0.3–0.9%, and the prevalence of TS, PTD, and CTD was 0.77, 2.99, and 1.61%, respectively (2). The prevalence of TD in boys was 1.06%, which was significantly higher than that in girls (0.25%) (3). TD is commonly associated with a variety of neuropsychiatric disorders, such as attention deficit hyperactivity disorder (ADHD), obsessive-compulsive disorder (OCD), anxiety disorders, mood disorders, autism, and sleep disorders and so on. There are a high proportion of individuals with TD (61–96%) have at least one comorbid psychiatric disorder. These comorbidities have a greater impact on the learning, working and social life of the sufferers than TD itself (4).

TD are common in children, however the underlying pathophysiology mechanism is still unclear. Many studies have focused on the disinhibition of the corticostriatal–thalamocortical circuit, genetic causes, environmental factors, immune factors and so on. Increasing evidence supports the involvement of infections, such as group A β hemolytic streptococcus (GABHS), which is closely associated with PANDAS (Neuropsychiatric Disorders Associated with Streptococcal Infections) (5). After GAS infection, PANDAS may develop into TD, obsessive-compulsive disorder, and emotional liability (6). In addition to streptococcal infections, other pathogens such as Mycoplasma pneumonia, Chlamydia trachomatis, Chlamydia pneumoniae and Toxoplasma are also considered as the possible causes of TD (7, 8). Present studies believed that infections could contribute to TD by triggering an immune response, which may influence the onset and the severity of TD. However, it still remains unclear whether TD is partly due to the infection or to the immune imbalance caused by an infection.

Autoimmune dysfunction has been proposed in the pathogenetic mechanism of many central nervous system (CNS) diseases. Immunologic factors are implicated in neurodevelopment, and their alteration could result in different disorders, such as tic disorders, developmental encephalopathies (9, 10), epilepsy (11), neuropsychiatric disorders (12), neurodegenerative diseases (13), genetic white matter disorders and other related diseases. Recent studies found that the meningeal lymphatic system is directly connected to the brain and the lymphatic system (14), which strengthens the interconnection between the nervous system and the immune response. Researchers indicated that dysregulation of the immune system may play an important role in TD. To date, many studies have been carried out to investigate whether cytokines associated with the innate immune response are altered under baseline conditions and during periods of symptom exacerbation. Leckman et al. found that the serum levels of IL-12 and TNF-α in children with tics at baseline were significantly higher than those in healthy controls. And during periods of symptom exacerbation, the concentrations of these two markers further increased (15). Gabbay et al. found that the distribution of cytokines in adolescents and children with TD were different. IL-12 was significantly higher in the TD group than that in the healthy control group. Moreover, the level of IL-2 in the TD with OCD group was significantly higher than that in the TD without OCD group, which indicated that IL-2 may be a biomarker to distinguish the difference between TD and its comorbidities (16). In addition to IL-2, Cheng et al. also found that IL-1 β, IL-6 and IL-17 in the TS group were significantly higher than those in the control group (17). Bos-Veneman et al. showed that IL-2 was positively correlated with tic severity ratings, and IL-12 negatively with severity ratings of obsessive-compulsive symptoms (18).

To investigate whether cytokines associated with the innate immune response were altered under different status of TD, we collected and analyzed a large sample of serum cytokines, including IL-2, IL-4, IL-6, IL-10, TNF-α, and IFN-γ. Combining these results may help us understand the pathogenic mechanisms of TD.

## Materials and Methods

### Subjects

Children with tic disorder were recruited from Department of Neurology, Children's Hospital, Zhejiang University School of Medicine, from October 2017 to May 2020. The recruited children, aged between 1 year and 17.5 years old, with or without medication, were diagnosed as tic disorder, meeting all the criteria of DSM-V-TR.

The exclusion criteria were as followings: Have taken drugs that affect immune function (except psychotropic drugs) in the past 6 months, any immune or hematological diseases, any infectious diseases (including the common cold) that occurred in the previous month, Sydenham's chorea, bipolar disorders, major depression, mania, generalized developmental disorders, psychotic disorders, epilepsy, organic brain diseases, and serious medical diseases.

The control group was recruited from the same hospital during the routine physical examination, and they did not have other mental or inflammatory diseases, nor meet any current or past DSM-V-TR criteria for mental illness.

This study was approved by the ethics review committee of the Children's Hospital, Zhejiang University School of Medicine, National Clinical Research Center for Child Health, and obtained the informed consent of the children's parents or guardians.

### Clinical Assessments

The assessment was conducted by well-trained clinicians with experience in pediatric tic disorders. Yale Global Tic Severity Scale (YGTSS) (19) was used to assess the severity of tics. According to previous studies (20, 21), we divided the patients into three groups, minimal tics (YGTSS score, from 1 to 9), mild tics (YGTSS score, from 10 to 19), and moderate to severe tics (YGTSS score, >19).

### Cytokine Assay

Blood samples were collected after outpatient treatment. One ml blood sample was transferred to serum separation tube, centrifuge at 1,000 g and 20°C for 20 mins after coagulation. Serum was carefully collected and stored at 2–8°C before analyzed within 12 h. As described in the previous literature, the concentrations of IL-2, IL-4, IL-6, IL-10, TNF-α, and IFN-γ in the serum were measured quantitatively using cell bead array CBA (Cytometric Beads Array) human Th1/Th2 cytokine kit II (BD Biosciences, San Jose, California, USA), as described in the previously literature (22). The detection range of the six cytokine was from 1.0 to 5,000 pg/ml.

### Statistical Analysis

All values of patients and controls are expressed as mean ± SD. SPSS Statistics 23 was used to perform Mann-Whitney U test. The significance level was set as *p* < 0.05. Graphpad prism statistical software was used to make the figures.

## Results

### Description of Study Subject

We strictly followed the inclusion and exclusion criteria, and finally 1,724 people were included in the patient group. Information about gender, age, disease classification, severity of symptoms and previous medication for the tic patients are shown in the Table 1. The admitted children with tic disorder were composed of 1,527 boys and 197 girls, with a median age of 7.7 years, ranging from 1 to 17.5 years. Most of the TD children are 6–12 years old and are in the school age. The average age of onset of these children with TD is 6.5 years old, ranging from 1 year to 16.5 years old. According to our analysis, there is no significant correlation between the age of onset and the concentration of serum cytokines (data not shown). The average YGTSS score was 5.0 ± 2.3 in the minimal tic symptom group, 13.7 ± 2.5 in the mild tic symptom group, comparing with 26.4 ± 5.4 in the moderate and severe tic symptom group. About half of the patients were treated with drugs (785 of 1,724; 45.53%), including aripiprazole, haloperidol, clonidine, tiapride and nitrazepam. Patients under medication used one of these drugs or a combination of two or more drugs. The healthy control samples were taken from 550 normal children (306 boys and 244 girls), with a median age of 6.5 years, ranging from 6 months to 14.5 years at the time of routine physical checkup without any evidence of infection.

**Table 1:** Demographic features and clinical characteristic of patients.

**Variable**	**Total (*n* = 1,724)**	**Rate (%)**
**Gender**		
Male	1,527	88.57
Female	197	11.43
**Age (years)**		
<6	464	26.91
6–12	1,147	66.53
12–18	113	6.55
**Subgroup**		
Provisional tic disorder	723	41.94
Chronic motor or vocal tic disorder	414	24.01
Tourette syndrome	587	34.05
**YGTSS values**		
YGTSS value (1–9)	439	25.46
YGTSS value (10–19)	727	42.17
YGTSS value (>19)	558	32.37
**Medication**		
Medicated	785	45.53
Unmedicated	921	53.42
Unknown	18	1.04

### The Changes of Plasma Pro-inflammatory Cytokines in TD Patients

**Figure 1:**
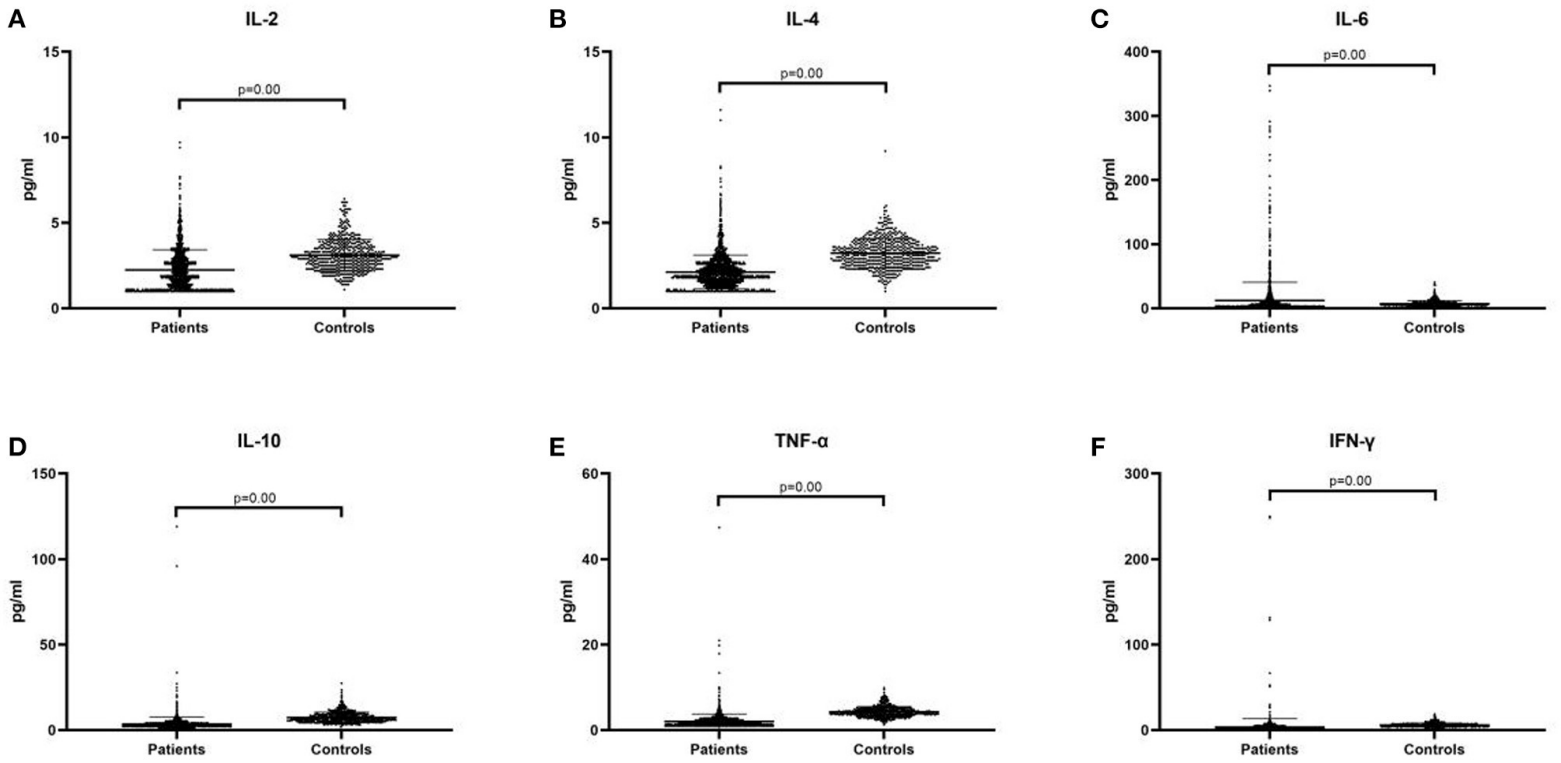
The expression of peripheral blood cytokines in the patient group and the control group. **(A)** IL-2. **(B)** IL-4. **(C)** IL-6. **(D)** IL-10. **(E)** TNF-α. **(F)** IFN-γ. We display the experimental data as mean ± SD value and *p* value.

**Figure 2:**
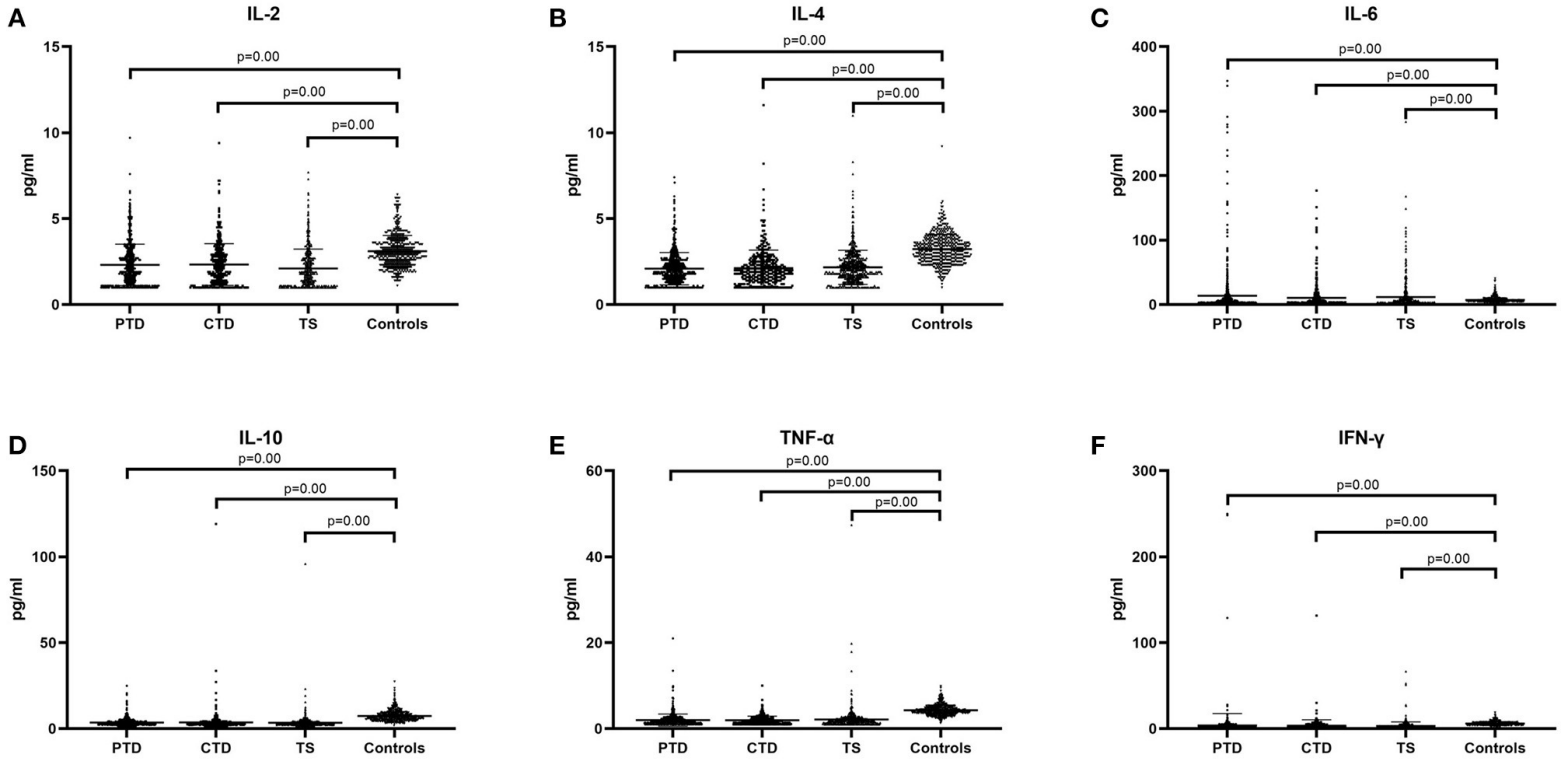
Concentration of cytokines in three diagnostic category groups of tic disorder and control group. PTD, provisional tic disorder; CTD, chronic motor or vocal tic disorder; TS, tourette syndrome. **(A)** IL-2. **(B)** IL-4. **(C)** IL-6. **(D)** IL-10. **(E)** TNF-α. **(F)** IFN-γ. We use the mean ± SD and *p* value to express data.

The plasma pro-inflammatory cytokine levels were compared between the control group and patient group by CBA. Our data showed that the average of serum concentration of IL-2 (2.24 ± 1.18 vs. 3.10 ± 0.92, *p* = 0.00, Figure 1A), IL-4 (2.12 ± 0.99 vs. 3.22 ± 0.86, *p* = 0.00, Figure 1B), IL-10 (3.56 ± 4.19 vs. 7.40 ± 3.09, *p* = 0.00, Figure 1D), TNF-α (2.04 ± 1.74 vs. 4.29 ± 1.21, *p* = 0.00, Figure 1E) and IFN-γ (3.46 ± 10.07 vs. 6.01 ± 2.23, *p* = 0.00, Figure 1F) in the children with TD were significantly lower than those in the control group, while IL-6 (12.31 ± 28.53 vs. 7.34 ± 4.73, *p* = 0.00, Figure 1C) was significantly higher than that in the control group. When comparing the provisional tic disorder group, chronic motor or vocal tic disorder group and Tourette syndrome group with the control group respectively, the average serum level of IL-2 (*p* = 0.00, Figure 2A), IL-4 (*p* = 0.00, Figure 2B), IL-10 (*p* = 0.00, Figure 2D), TNF-α (*p* = 0.00, Figure 2E) and IFN-γ (*p* = 0.00, Figure 2F) in these three groups of tics were significantly lower than that of the control group, while the average concentration of IL-6 (*p* = 0.00, Figure 2C) was significantly higher. Our results showed that the serum concentration of IFN-γ in female was lower than that in male, while no difference between genders was found for the other cytokines. In addition, there was no difference among ages was found for these six cytokines (data not shown).

### The Changes of Cytokines in Different Severity of Tic Disorder

**Figure 3:**
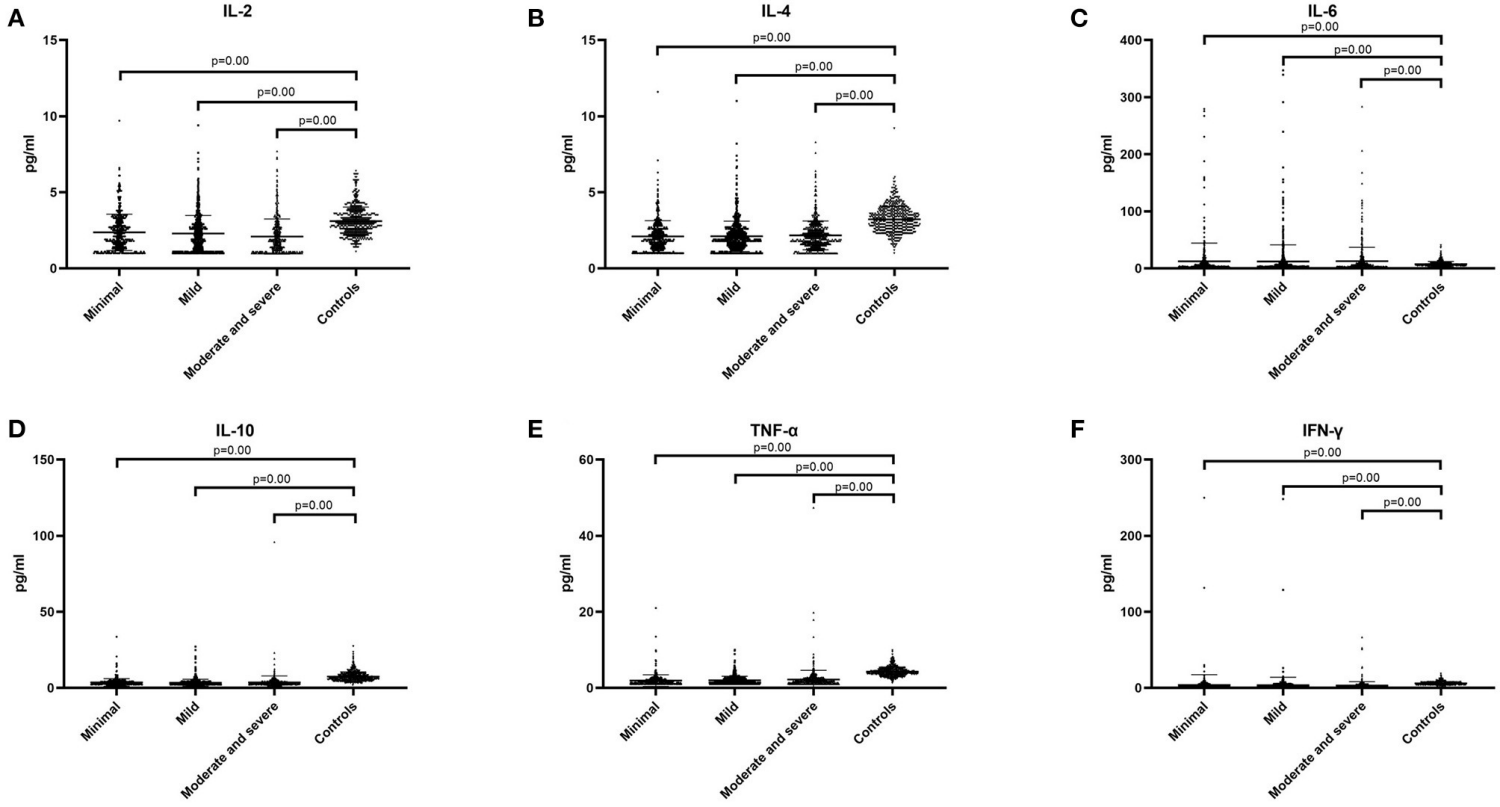
The concentration of six cytokines in three tic disorder groups of different severity and control group. Minimal means that YGTSS score is from 1 to 9. Mild means that YGTSS score is from 10 to 19. Moderate and severe means that YGTSS score is great than 19. **(A)** IL-2. **(B)** IL-4. **(C)** IL-6. **(D)** IL-10. **(E)** TNF-α. **(F)** IFN-γ. We use the mean ± SD and *p* value to express data.

We hypothesized that there may be statistical difference between levels of cytokines and severity of illness, so we divided the patients into three groups, including minimal tics group (YGTSS score, from 1 to 9), mild tics group (YGTSS score, from 10 to 19), and moderate to severe tics group (YGTSS score, >19). Comparing these three groups with the control group respectively, we found that in these three groups (minimal tics group, mild tics group, moderate to severe tics group), the concentrations of IL-2 (*p* = 0.00, Figure 3A), IL-4 (*p* = 0.00, Figure 3B), IL-10 (*p* = 0.00, Figure 3D), TNF-α (*p* = 0.00, Figure 3E) and IFN-γ (*p* = 0.00, Figure 3F) were significantly lower than those in the control group, while the concentration of IL-6 (*p* = 0.00, Figure 3C) was significantly higher than that in the control group.

### The Effects of Medication on the Serum Cytokines Concentration

Among the 1,724 patients, 785 children received drug treatment, 921 children did not receive any drug treatment before enrollment, and 18 children had unknown medication history. In the minimal tic symptom group, there were 126 patients who used drugs and 311 patients who did not use drugs. We found that the serum levels of IL-4 (2.23 ± 0.97 vs. 2.05 ± 1.05, *p* = 0.11), IL-10 (4.74 ± 18.36 vs. 3.75 ± 2.86, *p* = 0.04), IFN-γ (5.51 ± 22.21 vs. 3.12 ± 7.83, *p* = 0.00) of medication group were significantly higher than those of unmedication group, while the other three cytokine concentrations were not significantly different between the two groups (*p* > 0.05) (Table 2). In the mild tic symptom group, there were 345 patients who used drugs and 373 patients who did not use drugs. The two cytokines were found to be significantly different between the two groups. The concentration of IL-6 (8.75 ± 15.40 vs. 14.65 ± 36.89, *p* = 0.01) and IFN-γ (3.23 ± 2.61 vs. 3.70 ± 14.44, *p* = 0.01) in the medication group was significantly lower than those in the unmedication group, but the other four cytokines had no significant difference between the two groups with or without medication (*p* > 0.05). In the moderate and severe tic symptom group, there are 314 patients who used drugs and 237 patients who didn't. Serum concentrations of IL-2, IL-4, IL-6, IL-10, TNF-α, and IFN-γ were not found to be significantly different between the two groups (*p* > 0.05).

**Table 2:** Comparison of cytokines in three diagnostic category groups with or without medication.

			**IL-2**	**IL-4**	**IL-6**	**IL-10**	**TNF-α**	**IFN-γ**
			**(pg/ml)**	**(pg/ml)**	**(pg/ml)**	**(pg/ml)**	**(pg/ml)**	**(pg/ml)**
Minimal tics	Medicated	Mean ± SD	2.35 ± 1.23	2.23 ± 0.97	7.84 ± 13.59	3.15 ± 1.28	1.85 ± 0.76	5.51 ± 22.21
	Unmedicated	Mean ± SD	2.38 ± 1.18	2.05 ± 1.05	13.99 ± 35.89	3.75 ± 2.86	1.95 ± 1.74	3.12 ± 7.83
		*p*-value^*^	0.80	0.03	0.11	0.04	0.07	0.00
Mild tics	Medicated	Mean ± SD	2.27 ± 1.13	2.12 ± 0.97	8.75 ± 15.40	3.35 ± 2.16	1.91 ± 0.92	3.23 ± 2.61
	Unmedicated	Mean ± SD	2.31 ± 1.24	2.09 ± 1.02	14.65 ± 36.89	3.75 ± 6.41	2.07 ± 1.16	3.70 ± 14.44
		*p*-value^*^	0.95	0.40	0.01	0.22	0.28	0.01
Moderate and severe tics	Medicated	Mean ± SD	2.07 ± 1.11	2.11 ± 0.83	11.86 ± 24.85	3.45 ± 2.02	2.30 ± 3.11	2.90 ± 3.58
	Unmedicated	Mean ± SD	2.10 ± 1.21	2.22 ± 1.06	13.69 ± 24.08	3.70 ± 6.28	2.01 ± 1.20	3.52 ± 6.24
		*p*-value^*^	1.00	0.55	0.93	0.64	0.21	0.69

*Minimal tics (YGTSS score, 1–9), Mild tics (YGTSS score, 10–19), and Moderate to severe tics (YGTSS score, >19). SD, standard deviation*.

* *Mann–Whitney U Test*.

## Discussion

Current studies have showed that the abnormal immune response caused by infection may be the underlying pathological mechanisms of TD. It has been investigated to see if there is a relationship between TD and inflammatory cytokines. Consistent with the results from the previous studies, there is a certain relationship between the concentration of cytokines and the onset of tic disease. It has been shown that increased concentrations of MCP-1 and IL-2 in the basal ganglia, demonstrating the inflammatory response in the central nervous system of TS (23). Moreover, the concentrations of these pro-inflammatory cytokines in serum may represent the severity of tic symptoms in the TD patients. The study by Leckman et al. showed that during symptom deterioration in TS children, serum IL-12 and TNF-α concentrations were increased (15). Another study similarly concluded that 70% of patients expressed higher concentration of TNF-α when their tic symptoms were aggravated (24). Of course, the use of drugs may also have a certain influence on the concentration of cytokines. It has been shown that TNF-α and IL-13 concentration were increased in the children receiving antipsychotics when the tic symptoms exacerbated, while serum IL-4 concentration was increased in children receiving antipsychotics when the symptoms relieved (24), but another study showed that serum TNF-a concentration did not show any differences between medicated and non-medicated TS patients (25). However, whether these cytokines are involved in the pathogenesis of TD remains to be determined. To further investigate the role of cytokines and TD development in children, we examined related plasma cytokine concentrations in both the patient and control groups, and analyzed whether antipsychotics had any effects on the cytokines. Among the six cytokines, except that the serum concentration of IL-6 in patients was higher than that in the control group, the concentrations of the other five cytokines were significantly lower than that in the control group. The elevated serum IL-6 concentration in the patient group was consistent with only parts of previous reports (17, 26), whereas several other studies reported no changes of IL-6. Like these two studies, the participants were Asian children, which may suggest an ethnic influence on cytokines. IL-6 has both pro-inflammatory and anti-inflammatory properties and plays an important role in acute infection. When it binds to the soluble receptor sIL-6R, it affects the transition from acute inflammation to chronic inflammation by changing the nature of leukocyte infiltration (27). There are also many studies showing that IL-6 plays a key role in the pathogenesis of many immune diseases and chronic diseases, such as rheumatoid arthritis (28), idiopathic arthritis (29), diabetes (30), asthma, etc. After the treatment of rheumatoid arthritis and asthma with blocking IL-6, inflammatory activity was significantly suppressed (31, 32). IL-6 is involved not only in the regulation of immune response, but also in the initiation of signal transduction in complex neurophysiological functions such as mood, sleep, memory, and neuroimmune regulation. It is worth mentioning that IL-6, as a multidirectional cytokine, plays a key role in the interaction between the immune and nervous systems. Changes in IL-6 are found in some neurological diseases. For example, IL-6 is considered to be a key mediator of neuromyelitis optica (33). In animal experiments, it was shown that IL-6 increased the activity of serotonin (5-HT) and dopamine in the hippocampus and prefrontal cortex, and mice treated with IL-6 showed more frequent digging, feeding, and grooming behaviors (34, 35). In our study, the concentration of TNF-α is lower than that of the control group, which is consistent with the results of study by Matz et al. (25), but is different from the results of the other two studies (15, 26). This may be related to different methodology in different labs. Most patients in our study and Judith Matz et al.'s study had not been treated with medication, and the patients who used drugs are basically dopamine receptor antagonists. But in Leckman et al.'s study, patients were mainly treated with alpha agonists and selective serotonin reuptake inhibitors. Studies have shown that the use of certain psychotropic drugs, such as haloperidol and risperidone, can cause an increase in the concentration of TNF-α (36). The level of IL-2 in children with TD is lower than that in the control group. It is noteworthy that the dopaminergic system may be involved in the mechanism of tic disorders, and a study have shown that IL-2 can enhance the release of dopamine (37). And the results of an animal experiment indicated that the long-term changes in the stereotype of adolescent mice are related to the IL-2 mechanism, which may be the use of dopamine (38). The relationship between IL-2 and the severity of tic disorder may indicate that cellular immunity is involved in tic disorder. Our data showed that the concentrations of IL-4 and IL-10 in children with TD are also lower than those in the control group, which is inconsistent with the previous studies (15, 18). The difference in these results may be related to the factors, such as age, race, drug therapy and the presence of comorbidities.

Through the analysis of TD patients with and without drugs, we found that in the minimal tic syndrome group, the serum concentrations of IL-4, IL-10, and IFN-γ in the medication group were significantly higher than those in the unmedication group. In the mild tic symptom group, the concentrations of IL-6 and IFN-γ in the medication group were significantly lower than those in the unmeditation group. But in the moderate and severe tic symptom group, we did not find a significant difference in the concentrations of these six cytokines between the medication group and the unmedication group. These observations indicate that cytokine levels may be affected by drug status. A study have shown that levels of IL-4, IL-10, and IFN-γ increase after treatment with antipsychotic drugs (39). This result is the same as the cytokine difference we found in the minimal tic symptom group, but we did not find the same results in the moderate and severe tic syndrome groups. It is possible to guess that the treatment effect of minimal tic disorder will be better than severe tic disorder, medication in the early course of the disease can alleviate the child's condition and achieve better the therapeutic effect. Moreover, the serum IFN-γ concentration in female was lower than that of male. This may be due to the fact that the number of female patients included was far fewer than that of males. In this study, we only recruited Asian Chinese children and adolescents, and the sample size was relatively large, with a total number of 1,724, which has research significance for Asian children with TD.

Tic disorder is affected by many factors, such as genetic susceptibility, environmental factors, and immune factors. In the current largest prospective cohort study, environmental factors, microbial antigen immune response and host immune regulation capacity, genes, and development of predictive models for the onset and aggravated risk of tic disorder are separately studied (40). Combined with our research content, future research should conduct regular follow-up of TD patients after treatment, deeply explore the judgment of cytokines concentration on the severity of the disease, and grasp the course of drug treatment, so as to guide medication and reduce recurrence.

Our study had several limitations. This study did not assess comorbidities such as obsessive-compulsive disorder or ADHD as previously reported, so it could not be compared with previous studies. Serum cytokines are affected by a variety of factors and cannot directly reflect the inflammation in the central nervous system. This is another limitation in our study. All of these above need to be further studied and explored.

## Data Availability Statement

The raw data supporting the conclusions of this article will be made available by the authors, without undue reservation.

## Ethics Statement

The studies involving human participants were reviewed and approved by the Ethics Review Committee of the Children's Hospital, Zhejiang University School of Medicine, and National Clinical Research Center for Child Health. Written informed consent to participate in this study was provided by the participants' legal guardian/next of kin.

## Author Contributions

PJ and TZ contributed to conception and design of the study. PJ organized the database. YT performed the statistical analysis and wrote the first draft of the manuscript. ZX and XT wrote sections of the manuscript. All authors contributed to manuscript revision, read, and approved the submitted version.

## Funding

This work was supported by Grants from the National Natural Science Foundation of China (Nos. 82171438, 81671287, 81372116, and 81201511), Health Innovative Talents in Zhejiang Province, and Zhejiang Province Public Welfare Technology Application Research Project (Nos. LY22H090005 and LY15H090006).

## Conflict of Interest

The authors declare that the research was conducted in the absence of any commercial or financial relationships that could be construed as a potential conflict of interest.

## Publisher's Note

All claims expressed in this article are solely those of the authors and do not necessarily represent those of their affiliated organizations, or those of the publisher, the editors and the reviewers. Any product that may be evaluated in this article, or claim that may be made by its manufacturer, is not guaranteed or endorsed by the publisher.
